# Supplementary material for: Enhancing antibody responses by multivalent antigen display on thymus-independent DNA origami scaffolds
Source: Nat Commun. 2024 Jan 30;15:795. doi: 10.1038/s41467-024-44869-0 (PMC10828404; doi:10.1038/s41467-024-44869-0)
Supplement: Supplementary file 1 — Supplementary Information [file 41467_2024_44869_MOESM1_ESM.pdf]

Supplementary Information

Title

Enhancing antibody responses by multivalent antigen display on thymus-independent DNA origami scaffolds

Authors

Eike-Christian Wamhoff[1, *], Larance Ronsard[2, *], Jared Feldman[2, *], Grant A. Knappe[1,3, *], Blake M. Hauser[2, *], Anna Romanov[1,4], James Brett Case[5], Shilpa Sanapala[5], Evan C. Lam[2], Kerri J. St. Denis[2], Julie Boucau[2], Amy K. Barczak[2], Alejandro B. Balazs[2], Michael S. Diamond[5,6,7], Aaron G. Schmidt[2,8, **], Daniel Lingwood[2, **], and Mark Bathe[1,9,10, **]

Affiliations

[1]Department of Biological Engineering, Massachusetts Institute of Technology, Cambridge, MA 02139, United States

[2]Ragon Institute of Massachusetts General Hospital, Massachusetts Institute of Technology and Harvard University, Cambridge, MA 02139, United States

[3]Department of Chemical Engineering, Massachusetts Institute of Technology, Cambridge, MA 02139, United States

[4]Koch Institute for Integrative Cancer Research, Massachusetts Institute of Technology, Cambridge, MA 02139, United States

[5]Department of Medicine, Washington University School of Medicine, St. Louis, MO 63110, United States

[6]Department of Molecular Microbiology, Washington University School of Medicine, St. Louis, MO 63110, United States

[7]Department of Pathology & Immunology, Washington University School of Medicine, St. Louis, MO 63110, United States

[8]Department of Microbiology, Harvard Medical School, Boston, MA 02115, United States

[9]Broad Institute of MIT and Harvard, Cambridge, MA 02139, United States

[10]Harvard Medical School Initiative for RNA Medicine, Harvard Medical School, Boston, MA 02115, United States

*These authors contributed equally

**Correspondence to: mark.bathe@mit.edu, dlingwood@mgh.harvard.edu, aschmidt@crystal.harvard.edu

Supplementary Notes

Supplementary Note 1 – Fabrication of DNA-VLPs

X-ray structures of the ACE2-RBD complex suggested that modifications at the C-terminus of the antigen would allow for recognition of neutralizing epitopes including the receptor binding motif (RBM)[1]. We cloned and expressed recombinant RBD bearing a C-terminal His-tag followed by an additional Cys using HEK Expi293F cells (RBD-Cys). Incubation of RBD-Cys with TCEP revealed that at least 1 of the 4 disulfide bridges was susceptible to reduction as determined by Ellman's assay (**Supplementary Figure 2**). To ensure selective labeling of the C-terminal Cys, the reduced antigen was purified into PBS containing 10 mM EDTA and incubated at room temperature to allow for reoxidation. After approximately 6 h, the number of reduced Cys stabilized at 1 per RBD, indicating kinetically favored reoxidation of the disulfide bridges over the C-terminal Cys (**Supplementary Figure 2**). The reduced RBD-Cys was reacted with an SMCC-azide linker to afford RBD-Az. Conversion of the linker reaction was validated by selective labeling of the azide with Cy5-DBCO and subsequent ratiometric absorbance measurements. In addition to flow cytometry experiments with ACE2-expressing HEK293 cells, the preservation of the RBM for RBD-Az was validated by biolayer interferometry. Reaction conditions for the functionalization of DBCO-bearing DNA-VLPs were screened and set to at least 150 nM DNA-VLP at 30 equivalents of RBD-Az per DBCO to achieve near-quantitative coverage. Following 24 h incubation at room temperature, **DNA-VLP-1x**, **-6x**, and **-30x** were purified by drop dialysis. Notably, functionalization efficiency was dependent on maximum DBCO concentrations rather than nanoparticle concentrations, and we were only able to obtain approximately 30% functionalization efficiency for **DNA-VLP-1x**.

Supplementary Figures

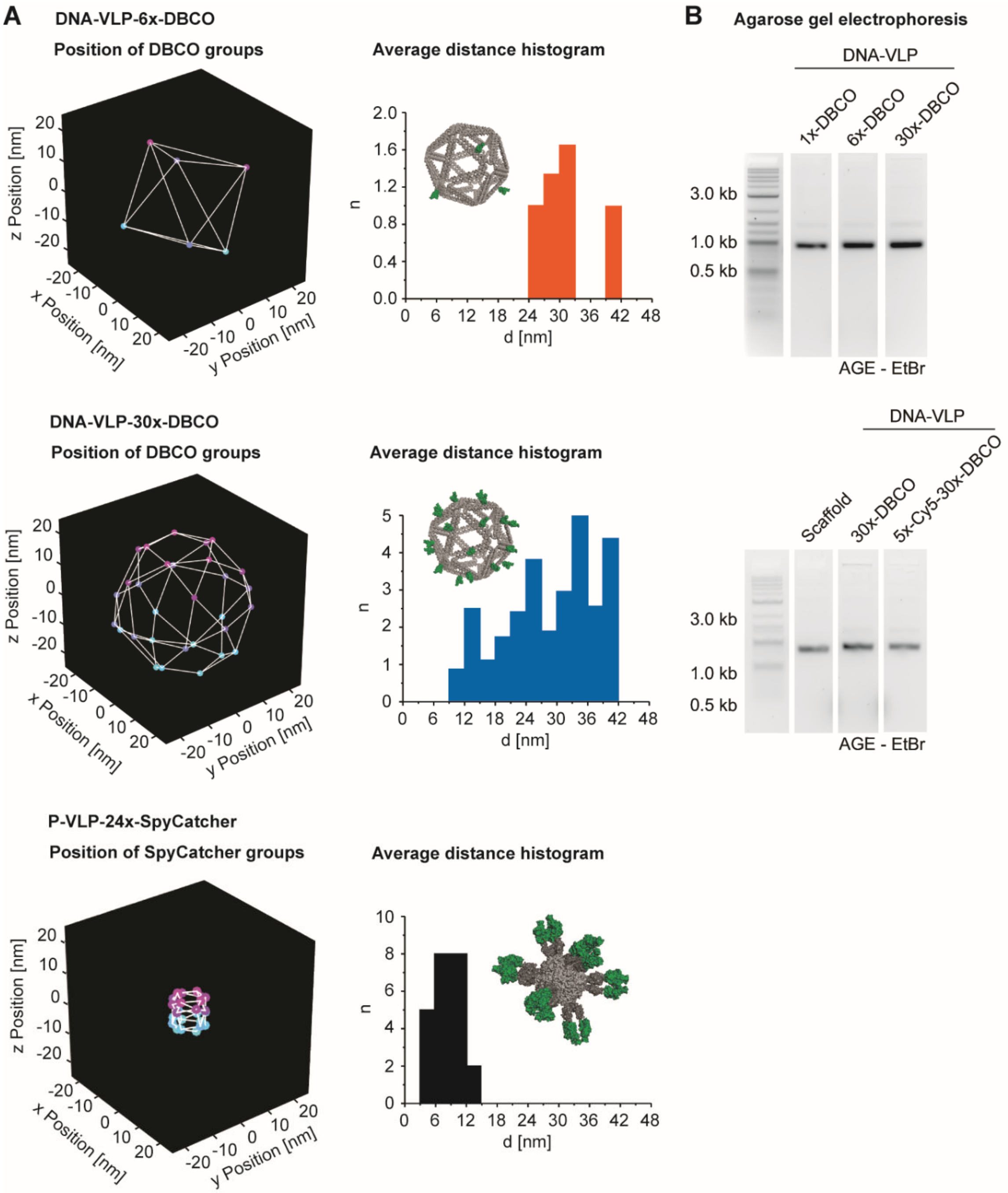

Supplementary Figure 1 – Supporting information for DNA-VLP and P-VLP design and assembly.

(A) The positions of functional groups were mapped by proxy of the 5'OH groups of modified staples for **DNA-VLP-6x-DBCO** and **DNA-VLP-30x-DBCO** and of the SpyCatcher locations for **P-VLP-24x-SpyCatcher**. These mapped positions served to calculate distance histograms, using the average, rank-ordered distances between each functional group and all neighboring conjugation sites. **(B)** Agarose gel electrophoresis served to validate the assembly of DBCO-functionalized DNA-VLPs. Images are representative from n=3 technical replicates.

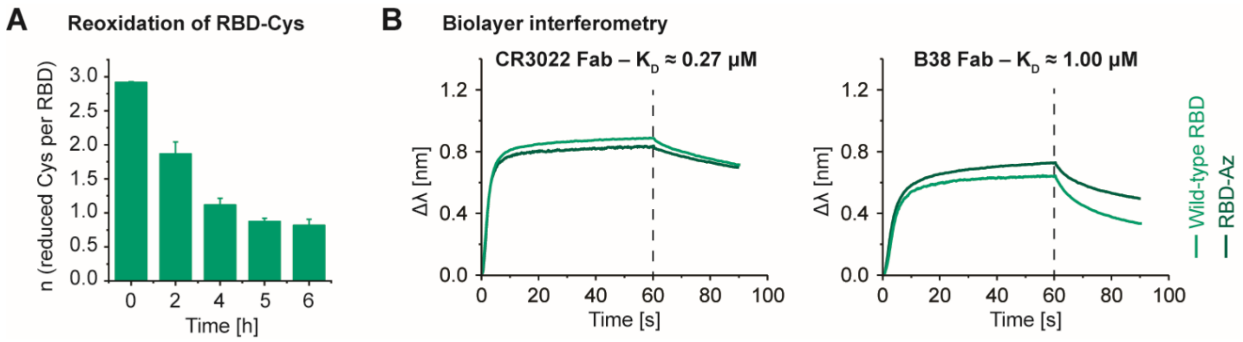

Supplementary Figure 2 – Supporting information for antigen expression and functionalization.

(A) The reduction and subsequent reoxidation of RBD-Cys in PBS with 10 mM EDTA was monitored by Ellman's assay. The reoxidation kinetics revealed that at least 1 of the 4 disulfide bridges was susceptible to reduction but reoxidation was favored compared to the additional, C-terminal Cys, allowing for selective modification with an SMCC-azide linker. Error bars represent the standard error of the mean. **(B)** In addition to flow cytometry experiments with ACE2-expressing HEK293 cells, the preservation of the RBM for RBD-Az was validated by biolayer interferometry at 10 µM antigen. Binding kinetics for both the CR3022 and B38 Fab fragments were comparable to wild-type RBD. All experiments were conducted with n=3 biological replicates. Representative biolayer interferometry curves are shown.

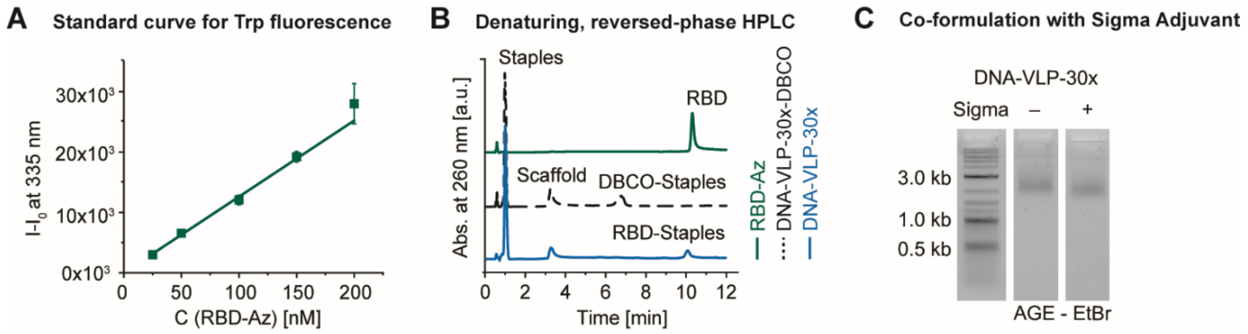

Supplementary Figure 3 – Supporting information for DNA origami functionalization.

(A) RBD coverage of DNA-VLPs was determined by Trp fluorescence as previously described for other antigens[2]. The corresponding standard curve for RBD-Az is shown. Trp fluorescence values were determined from n=3 biological replicates. Error bars represent the standard error of the mean. **(B)** Near-quantitative reaction conversion was further validated for **DNA-VLP-30x** via denaturing, reversed-phase HPLC[3]. **(C)** 100 nM **DNA-VLP-30x** were co-formulated with PBS (left) or Sigma adjuvant (right) at equal volumes and characterized by agarose gel electrophoresis (AGE), supporting their compatibility. Images are representative from n=3 technical replicates.

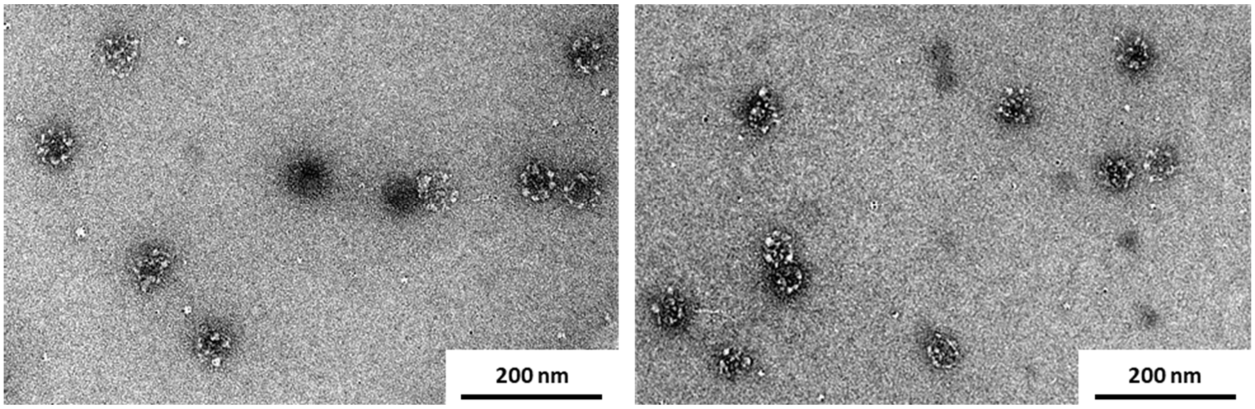

Supplementary Figure 4 – Supporting information for transmission electron microscopy.
Representative widefield transmission electron micrographs of **DNA-VLP-30x** are shown. 2% uranyl formate was used for negative staining. Images are representative from n=3 technical replicates.

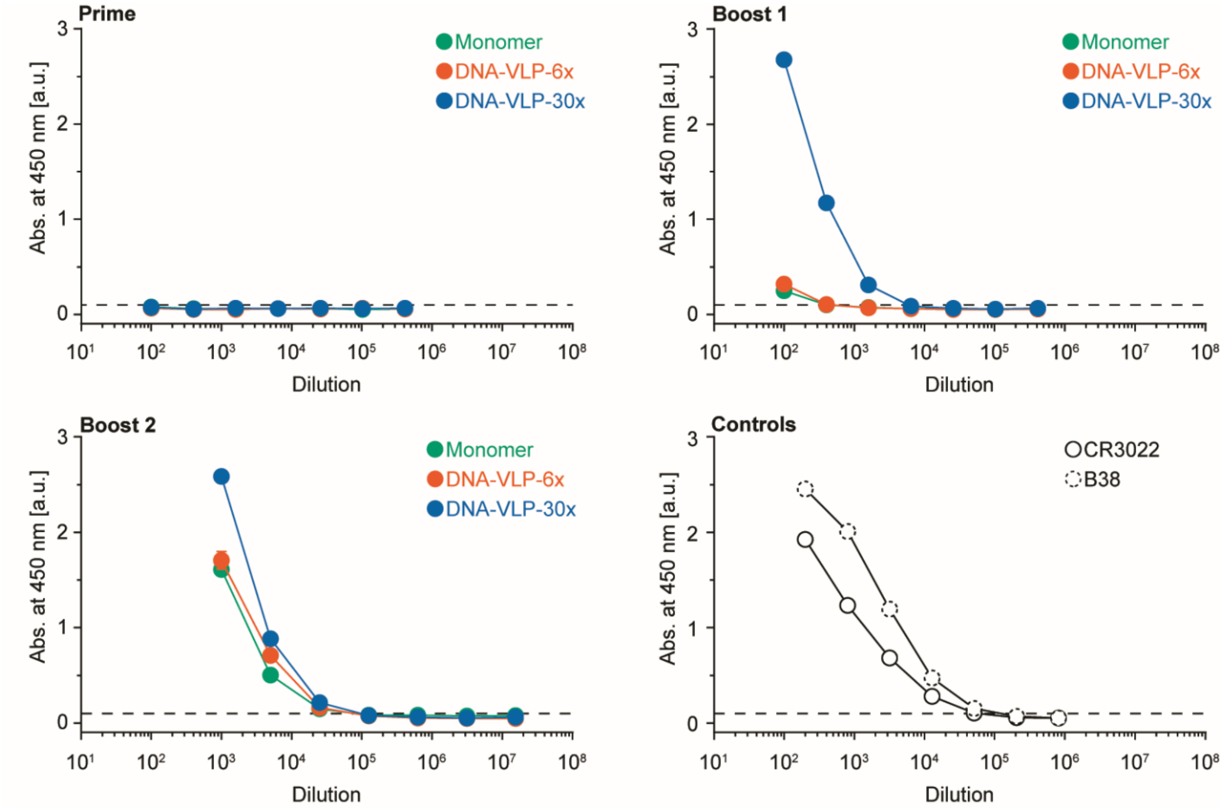

Supplementary Figure 5 – Supporting information for RBD-specific IgG ELISA.
Dilution curves for RBD-specific IgG ELISAs with mouse sera following immunization with 7.5 µg RBD are shown. Plates were coated with C-terminally cleaved RBD, devoid of 8x His and SBP tags. Absorbance values were determined from n=5 biological replicates. Error bars represent the standard error of the mean. Representative dilutions curves for RBD-specific control IgGs are shown from n=5 technical replicates.

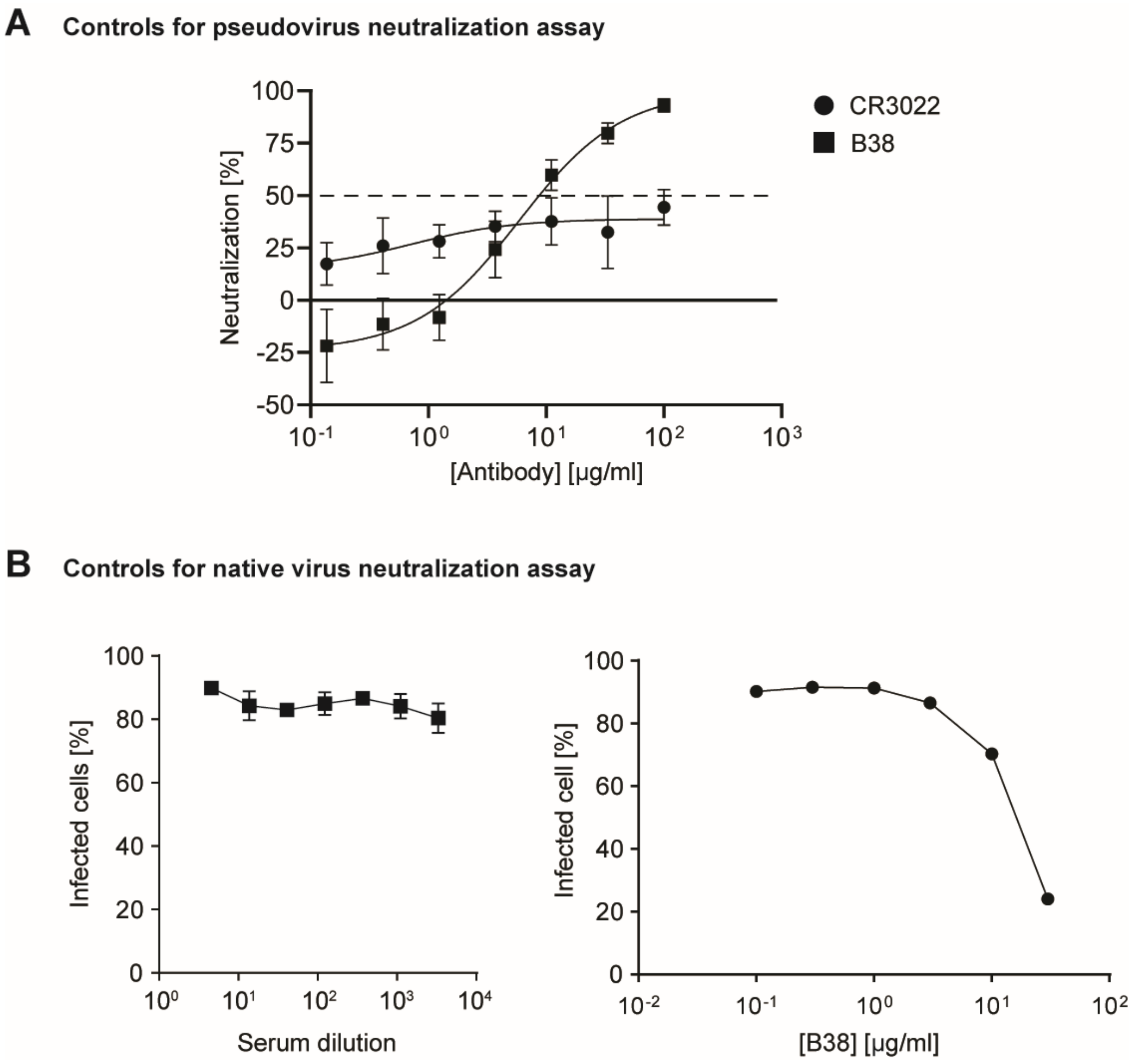

Supplementary Figure 6 – Supporting information for neutralization assays.
(A) Dose-response curves for positive (B38) and negative (CR3022) control IgGs for the pseudovirus neutralization assay. **(B)** Dose-response curves for negative (serum from naive mice, left) and positive (B38 IgG, right) controls in the native virus neutralization assay are shown. Error bars represent the standard error of the mean (n=5 biological replicates, n=1 biological replicate for B38 IgG control).

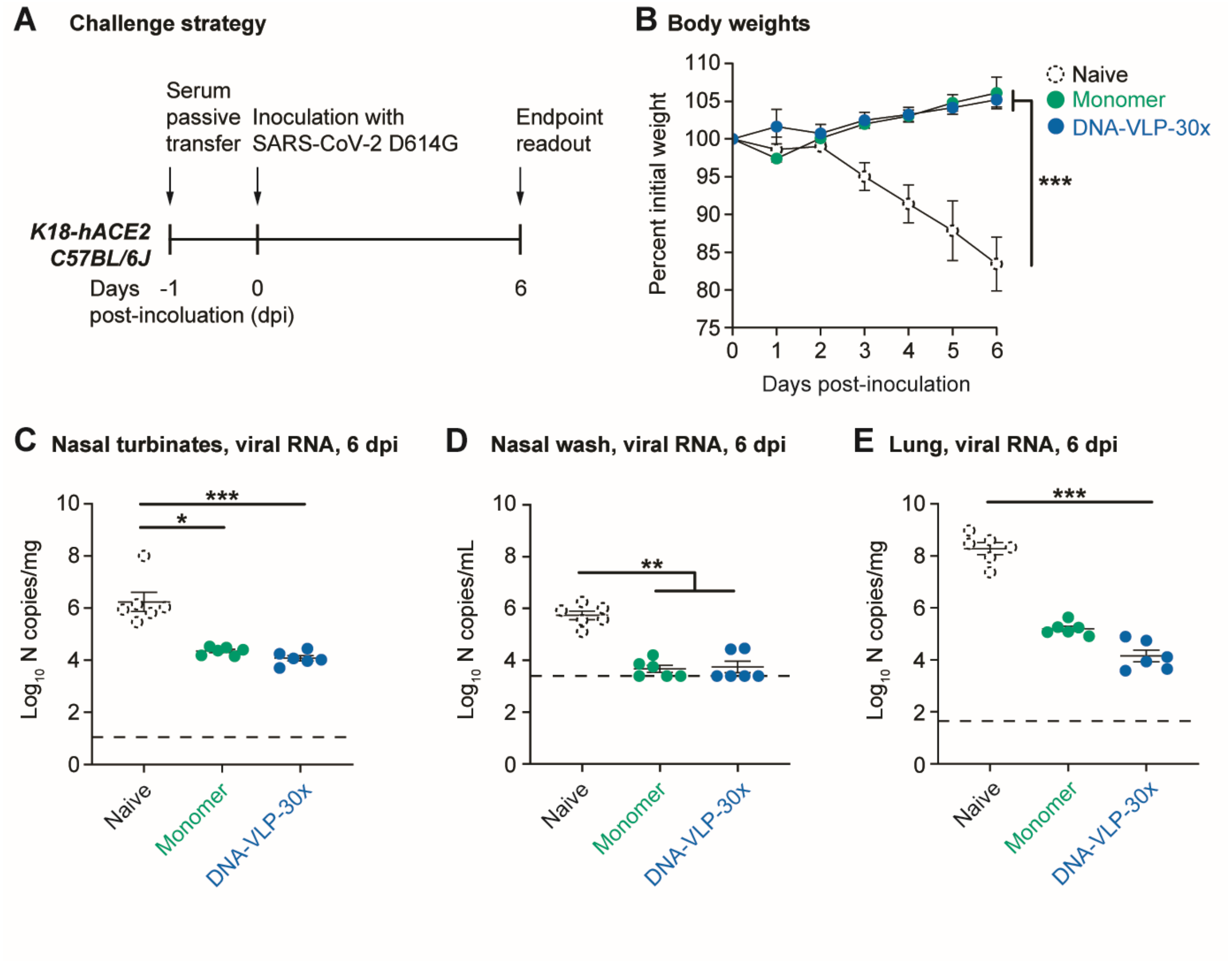

Supplementary Figure 7 – Supporting information for protection following passive transfer and SARS-CoV-2 challenge. **(A)** Passive transfer of immune sera followed by SARS-CoV-2 D614G live virus challenge. **(B)** Daily body weights of the three cohorts tested: immune sera from mice immunized with **DNA-VLP-30x** or monomeric RBD (post-boost 2 timepoint) and sera from non-immunized C57BL/6 as a control (n=6 female mice per group, error bars represent the standard error of the mean, ***P<0.0006, ANOVA with Dunnett's test of the area under the curve; p=0.0005 for Monomer, p=0.0002 for **DNA-VLP-30x**). **(C-E)** At day 6 post challenge tissues were harvested and viral RNA was measured in the nasal turbinates **(C)**, nasal washes **(D)** and in the lung **(E)** (n=6 mice per group, error bars represent the standard error of the mean, ***P<0.0001, **P<0.005, *P<0.05, Kruskal-Wallis test with Dunn's test corrected for multiple comparisons). For **(C)**, p=0.0463 for Monomer, p=0.0007 for **DNA-VLP-30x**. For **(D)**, p=0.0049 for Monomer, p=0.0099 for **DNA-VLP-30x**. For **(E)**, p=0.0002 for **DNA-VLP-30x**.

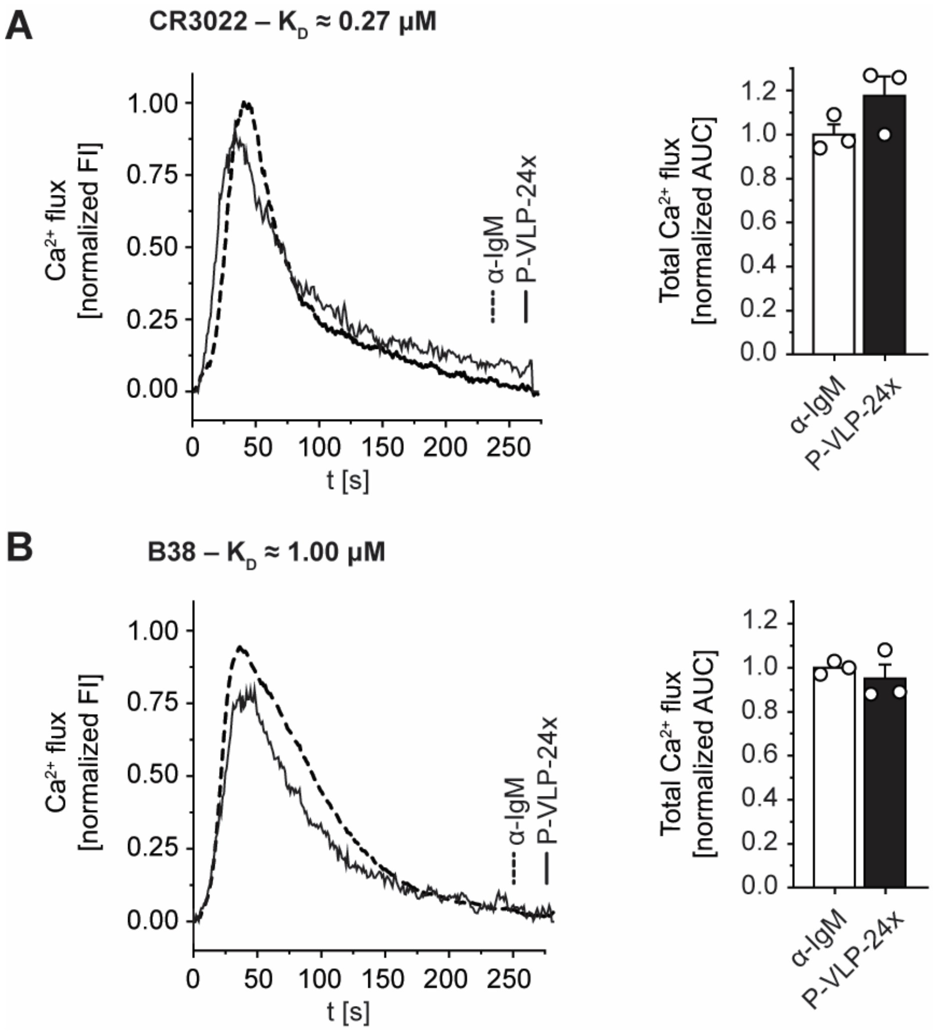

Supplementary Figure 8 – Supporting information for P-VLP B cell activation.

(A and B) Ramos B cells expressing the BCRs CR3022 and B38 were incubated with α-IgM and **P-VLP-24x** at 30 nM antigen. Ca^{2+} flux in response to RBD incubation was assayed using Fura Red. Representative fluorescence intensity (FI) curves are shown from n=3 biological replicates (left). Total Ca^{2+} flux was quantified via the normalized area under the curve (AUC) (right). Normalized AUCs were determined from n=3 biological replicates. Error bars represent the standard error of the mean.

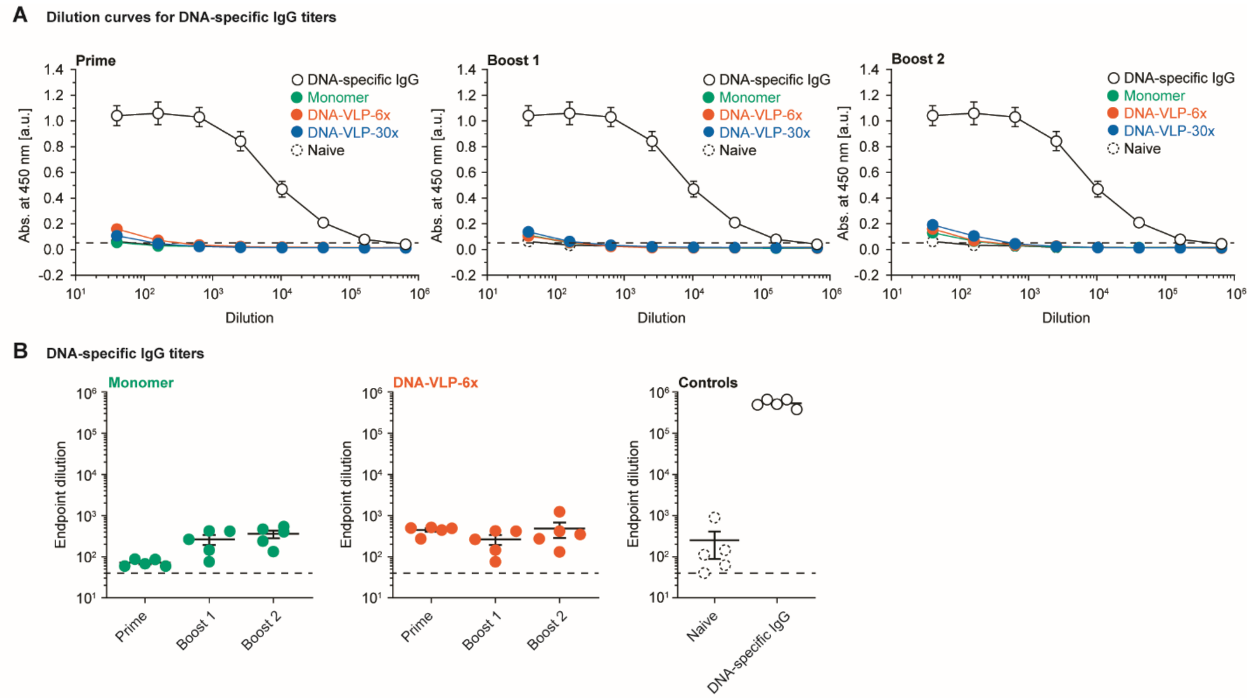

Supplementary Figure 9 – Supporting information for DNA-specific IgG ELISA

(A) Dilution curves for DNA-specific IgG ELISAs with mouse sera following immunization with 7.5 µg RBD are shown. Plates were coated with calf thymus DNA. **(B)** DNA-specific IgG endpoint dilutions for monomer RBD and **DNA-VLP-6x**. Naive serum and DNA-specific IgG served as the positive and negative controls for the ELISA, respectively. DNA-specific IgG was diluted from 10 µg/ml. Absorbance values and IgG titers were determined from n=5 biological replicates. One-way ANOVA was performed followed by Dunnett's T3 multiple comparison test at α=0.05. Error bars represent the standard error of the mean.

Supplementary Tables

Supplementary Table 1 – Scaffold sequence

GAGCGCAACGCAATTAATGTGCGCCCTGTAGCGGCGCATTAAGCGCGGCGGGTGTGGTGGTTACGCGCAGCG
TGACCGCTACACTTGCCAGCGCCCTAGCGCCCGCTCCTTTCGCTTTCTTCCCTTCCTTTCTCGCCACGTTCGCC
GGCTTTCCCCGTCAAGCTCTAAATCGGGGGCTCCCTTTAGGGTTCCGATTTAGTGCTTTACGGCACCTCGACCC
CAAAAAACTTGATTAGGGTGATGGTTCACGTAGTGGGCCATCGCCCTGATAGACGGTTTTTCGCCCTTTGACGTT
GGAGTCCACGTTCTTTAATAGTGGACTCTTGTTCCAAACTGGAACAACACTCAACCCTATCTCGGTCTATTCTTTT
GATTTATAAGGGATTTTGCCGATTTCGGCCTATTGGTTAAAAAATGAGCTGATTTAACAAAAATTTAACGCGAATT
ACAACCGGGGTACATATGATTGGGGTCTGACGCTCAGTGGAACGAAAACTCACGTTAAGGGATTTTGGTCATGA
GATTATCAAAAAGGATCTTCACCTAGATCCTTTTAAATTAAAAATGAAGTTTTAAATCAATCTAAAGTATATATGAG
TAAACTTGGTCTGACAGTTACCAATGCTTAATCAGTGAGGCACCTATCTCAGCGATCTGTCTATTTCGTTCATCCA
TAGTTGCCTGACTCCCCGTCGTGTAGATAACTACGATACGGGAGGGCTTACCATCTGGCCCCAGTGCTGCAATG
ATACCGCGAGACCCACGCTCACCGGCTCCAGATTTATCAGCAATAAACCAGCCAGCCGGAAGGGCCGAGCGCA
GAAGTGGTCCTGCAACTTTATCCGCCTCCATCCAGTCTATTAATTGTTGCCGGGAAGCTAGAGTAAGTAGTTCGC
CAGTTAATAGTTTGCGCAACGTTGTTGCCATTGCTACAGGCATCGTGGTGTCACGCTCGTCGTTTGGTATGGCTT
CATTCAGCTCCGGTTCCCAACGATCAAGGCGAGTTACATGATCCCCCATGTTGTGCAAAAAAGCGGTTAGCTCC
TTCGGTCCTCCGATCGTTGTCAGAAGTAAGTTGGCCGCAGTGTTATCACTCATGGTTATGGCAGCACTGCATAAT
TCTCTTACTGTCATGCCATCCGTAAGATGCTTTTCTGTGACTGGTGAGTACTCAACCAAGTCATTCTGAGAATAGT
GTATGCGGCGACCGAGTTGCTCTTGCCCGGCGTCAATACGGGATAATACCGCGCCACATAGCAGAACTTTAAAA
GTGCTCATCATTGGAAAACGTTCTTCGGGGCGAAAACTCTCAAGGATCTTACCGCTGTTGAGATCCAGTTCGATG
TAACCCACTCGTGCACCCAACTGATCTTCAGCATCTTTTACTTTCACCAGCGTTTCTGGGTGAGCAAAAACAGGA
AGGCAAAATGCCGCAAAAAAGGGAATAAGGGCGACACGGAAATGTTGAATACTCATACTCTTCCTTTTTCAATAT
TATTGAAGCATTTATCAGGGTTATTGTCTCATGAGCGGATACATATTTGAATGTATTTAGAAAAATAAACAAATAGG
GGTTCCGCGCACATTTCCCCGAAAAGTGCCACCTGACGTCTAAGAAACCATTATTATCATGACATTAACCTATAA
AAATAGGCGTATCACGAGGCCCTTTCGTCGAATTCGTCGTCGTCCCCTCAAACTCTTGGGTGGAGAGGCTATTC
GTTTAAGGTCACATCGCATGTAATTTACTTATTCTCTGTTGTTGAGCCACCCGGGCGCCAGATTTTGTTTAAAGCT
TTGTCTCTTAGTTTGTATAGACAGATTCAGAGTGCAAGGTTTCGTTCGCTCGTACCTGGTTTTCCCTGGTTCTTCA
CAGATAGGATTTGACTTTCTACAACACTTATGCGGCTTCCTACCCGTTTGAAGGCCGATACAGGTGCTGCGCAAA
ATGCGGGCGAACATAGAGTATCAAAACAACGCCTTCTAATCTAGGAATATAGGGAAGATACGTATTTGCTACCAT
GCTTTCTTGGGTCATTAACGACCAACCTCTTTTCTTTTAAAGTAGGATTGCACAATGAATGAATACACGTGGTCCG
ATAACTGACCAAGTAACATGGTTATCACTAGATGTCCGCCAGACGTGTGCAAACCAACCCGGGAGTTACGTCAC
TAATCCTTCGCTACGTCGTGAAGATATTTACTTGTGAATATCGAGGGTAATAAGATAATAGACTGTGACTAGTATT
GCCAGACTGTCGCTACCTGCAACACATAACTATCCTGAGGTTACTGCATAGTACTGATTACACCCGAGTCAAAAT
TTCTAACTTCTAACATGTACCTAGTAACCAGCTCAATAATTATGTCAGAATATAGCTCTGGGAACCCTCGGACAAT
TATGATACACGGTATTAATATCTTGCTTGCGTTAGCCACTTCTCATCTTTGGATACCGATTCTATTTTGCATAGCAG
TTCCTTTTACACATATAAGAATTTCGCCATAGGTATGCTTAAGGAAGTCGAGATTGCGAACCATTACCGAGACTAT
GGCTTCATGTGGTGATTTCACCCGACCCACCCTTGGCGCCAGCTTTACGCAGCTTCCTGACGATACGTGGTGTA
ACGTTGTGTTTGGCAATGGAAACCGAGATCAACTATTTCTAATGCTGATATAGCAGAGTCTCGCGTCTATCATAC
GCAAGTCGCACGTCATTTTCGAGAGCAGCGTAAGACTCTGAAGGTCATGAGCCCAGATGTTATTACCCTCTACCT
ATAAACATCAAAATTGTAGTCGTTTTACAGTCCATCGTCGCTCCAGAGCGAAGATTAAGGTTAGATCTAGATTATC
TTTGCACGTGTGGACCGACGCAGCTGGGGCTCTAGCTCCACTACGGTTACGAAACTGCTGAACGATCTGGTCCA
CTTCAAGATTCACACATCGTTTCATTCTTTGGACAACCAACACTCTCAGTCAGAGTTTCGAGTATAATAATTCTTCC
GCGCTAGGGTAAAAAGCAGATATGGGGAGACATTCCGGGCTTTTGAGCCGATACACTAAGCACTTGACATACTC
ACATCAGTAGAGGTTAACATTCATGACTATCACGCGCTGCAG

Supplementary Table 2 – Staple sequences

Staple number	Sequence
2	ACATCTGGATGGCGAAATTCTTATATGTGGTAGAGGGTAATA
3	GCTCATGACCAAGCATACCT
4	GTTCGCAATCTTTTTTCGACTTCCTTTTCAGAGTCTTTTTTTACGCTGCTCT
5	CGGTAATGTCACCACATGAAGCCGTCCGAGG
6	GTTCCCAGACCGTGTATCATAATTATAGTCT
7	GCGAGACTCTGTTTTTCTATATCAGCCCAAACACAACTTTTTGTTACACCACCCAAGGGTG GGTTTTTTCGGGTGAAA
8	AGCTGGCGGTATCGTCAGGAAGCTAACCTCA
9	GGATAGTTAATCAGTACTATGCAGTGCGTAA
10	TTCCATTGATTAGAAATAGTTGAGTAGCGAA
11	GGATTAGTTAAATATCTTCACGACTCTCGGT
12	TGATAGACCGAAAATGACGTGCGACACGTGC
13	AAAGATAAAGCTGCGTCGGTCCACTTGCGTA
14	CTACAATTTTGTTTTTATGTTTATAGTAAAAGGAACTTTTTTGCTATGCAAATTTTTATAGGT TTTTTTAATGTCATG
15	GTCAAGTGCGACGATGGACTGTAAAACGACTGATGTGAGTAT
16	CTTAGTGTATCGCTCTGGAG
17	TCTAGATCTAATTTTTCCTTAATCTTCGGCTCAAAAGTTTTTCCCGGAATGT
18	AGAATTATTATTTTTTACTCGAAACTGAAACGATGTGTTTTTTGAATCTTGACCGTAGTGGA GTTTTTCTAGAGCCCC
19	TTTCGTAAAGTGGACCAGATCGTACATCTAG
20	TGATAACCTGCACACGTCTGGCGGTCAGCAG
21	CAAAGAATCTGACTGAGAGTGTTTGATAACA
22	CTGCGGCCCTGCCATAACCATGAGGGTTGTC
23	AGCGCGGACTCCCCATATCTGCTTACCCGCC
24	GCGCTTAAGCGCGTAACCACCACTTTACCCT
25	CAACGTCAAAGTTTTTGGCGAAAAACAGTCATGAATGTTTTTTTAACCTCTA
26	GATGGCCCTGCGCTCCTGCAGCGCGTGATCGTCTATCAGGGC
27	ACTACGTGAATTAATTGCGT
28	TGCGCCGCTACTTTTTAGGGCGCACACCATCACCCTATTTTTATCAAGTTTT
29	GAACCCTAAAGTTTTTGGAGCCCCCGCGTGGCGAGAATTTTTAGGAAGGGAAGGCAAGTG TAGTTTTTCGGTCACGCT
30	AGGGCGCTGAAAGCGAAAGGAGCTAACCGCT
31	TTTTTGCAGGAGGACCGAAGGAGCGGGCGCT
32	CCGGCGAAATTTAGAGCTTGACGATCATATG
33	TACCCCGGTGAGCGTCAGACCCCAGGGAAAG
34	CTAAATCGTTGGGGTCGAGGTGCCAGACCGA
35	GATAGGGTATAAATCAAAAGAATGTAAAGCA
36	CTTAGACGACTATTAAAGAACGTGGACTCATAATAATGGTTT
37	TCAGGTGGCAACAAGAGTCC
38	TGAGTGTTGTTTTTTTCCAGTTTGGACTTTTCGGGGATTTTTAATGTGCGCG
39	ATTCAAATATGTTTTTTATCCGCTCACGTAGTTATCTTTTTTACACGACGGGCCGAAATCGG CTTTTTAAAATCCCTT

40	ATGGATGAGCTCATTTTTTAACCAATAGGGAGTCAGGCAACT
41	ACGAAATAGATGTTAAATCA
42	TAACGTGAGTTTTTTTTTCGTTCCACTTGTAATTCGCTTTTTGTTAAATTTTCAGATCGCTGA TTTTTGATAGGTGCC
43	AAATCCCTCTTTTTGATAATCTCGAACCGGA
44	GCTGAATGTCGCCTTGATCGTTGGATGACCA
45	CAAGTTTACTCTTTTTATATATACTTAAAAGGATCTATTTTTGGTGAAGATC
46	TTTAATTTTAGATTGATTTAAAACTAGCTTC
47	CCGGCAACTGGCGAACTACTTACTCTTCATT
48	TGTCAGACTCACTGATTAAGCATGGAGCCGG
49	TGAGCGTGTATTGCTGATAAATCTTGGTAAC
50	CTGATAAAAGATGGTAAGCCCTCCCGTATTGAGACAATAACC
51	TGCTTCAATACACTGGGGCC
52	GGTCTCGCGGTTTTTTATCATTGCAGATATTGAAAAATTTTTGGAAGAGTATCCTTTTTTGC GTTTTTGCATTTTGCC
53	TGAAAGTAAAATTTTTGATGCTGAAGGGCCCTTCCGGTTTTTCTGGCTGGTT
54	ACGAGTGGTGCAGGACCACTTATGCGCTCATCAGTTGGGTGC
55	GTTACATCGACGGATAAAGT
56	AATTAATAGACTTTTTTGGATGGAGGACTGGATCTCATTTTTACAGCGGTAA
57	TTCCAATGATGTTTTTAGCACTTTTAATTGACGCCGGTTTTTGCAAGAGCAAAACGTTGCG CATTTTTAACTATTAAC
58	CAATGGCAACCTCGGTCGCC
59	GCCTGTAGGCATACACTATTCTCAGAATGGTGACACCACGAT
60	CAACATGGGGGTTTTTATCATGTAACAAGCCATACCATTTTTAACGACGAGCACTTGGTTG AGTTTTTTACTCACCAG
61	CAGTAAGAGAATTTTTTTATGCAGTGAACTTACTTCTTTTTTGACAACGATC
62	TGGCATGATCACAGAAAAGCATCTCATTGTG
63	CAATCCTAGACCACGTGTATTCATTTACGGA
64	TATCCCGTAAGTTCTGCTATGTGGGAAAGCA
65	TGGTAGCATCGTTAATGACCCAACGCGGTAT
66	AGAACGTTGATCCTTGAGAGTTTTCTTCAAA
67	CGGGTAGGAGCACCTGTATCGGCCGCCCCGA
68	AACGCTGGTTCCTGTTTTTGCTCGTGAAGAA
69	CCAGGGAAAAGTCAAATCCTATCTACCCAGA
70	CCTTATTCGAGTATTCAACATTTCTGGCTCA
71	ACAACAGAAATCTGGCGCCCGGGCGTGTCGC
72	CTAAATACGAACCCCTATTTGTTTAGCCTCT
73	CCACCCAATGTGACCTTAAACGAATATTTTT
74	CCAAAGATAAGGGCCTCGTGATACGCCTAATAGAATCGGTAT
75	GAGAAGTGGCAATTCGACGA
76	GAATAAGTAAATTTTTTTACATGCGAGAGTTTGAGGGTTTTTGACGACGACG
77	TAACGCAAGCATTTTTAGATATTAATAGCTATATTCTTTTTTGACATAATTAAAGAGACAAAG TTTTTCTTTAAACAA
78	TAGGTACACTGAATCTGTCTATACAAACTTTGAGCTGGTTAC
79	TGTTAGAAGTACCTTGCACT
80	AAGCCGCATAATTTTTGTGTTGTAGAAACCAGGTACGTTTTTAGCGAACGAA

81	TAGAAATTTTGTTTTTACTCGGGTGTATGTGTTGCAGTTTTTGTAGCGACAGATGTTCGCC CGTTTTTCATTTTGCGC
82	TTGATACTCTTCTGGCAATA
83	GCGTTGTTCTAGTCACAGTCTATTATCTTCCTAGATTAGAAG
84	ATGTTACTTGGTTTTTTCAGTTATCGCTTTAAAAGAATTTTTAAGAGGTTGGAATACGTATCT TTTTTTCCCTATATT
85	ATTACCCTCGATTTTTTATTCACAAGGACGTAACTCCTTTTTCGGGTTGGTT

Supplementary Table 3 – Modified staple sequences

Cy5-modified staples

Staple number	Sequence
19	**/Cy5-TEG/TT**TTTCGTAAAGTGGACCAGATCGTACATCTAG
30	**/Cy5-TEG/TT**AGGGCGCTGAAAGCGAAAGGAGCTAACCGCT
46	**/Cy5-TEG/TT**TTTAATTTTAGATTGATTTAAAACTAGCTTC
69	**/Cy5-TEG/TT**CCAGGGAAAAGTCAAATCCTATCTACCCAGA
72	**/Cy5-TEG/TT**CTAAATACGAACCCCTATTTGTTTAGCCTCT

DBCO-modified staples – I52-1x-DBCO

Staple number	Sequence
9	**/DBCO-TEG/TT**GGATAGTTAATCAGTACTATGCAGTGCGTAA

DBCO-modified staples – I52-6x-DBCO

Staple number	Sequence
9	**/DBCO-TEG/TT**GGATAGTTAATCAGTACTATGCAGTGCGTAA
15	**/DBCO-TEG/TT**GTCAAGTGCGACGATGGACTGTAAAACGACTGATGTGAGTAT
32	**/DBCO-TEG/TT**CCGGCGAAATTTAGAGCTTGACGATCATATG
54	**/DBCO-TEG/TT**ACGAGTGGTGCAGGACCACTTATGCGCTCATCAGTTGGGTGC
64	**/DBCO-TEG/TT**TATCCCGTAAGTTCTGCTATGTGGGAAAGCA
71	**/DBCO-TEG/TT**ACAACAGAAATCTGGCGCCCGGGCGTGTCGC

DBCO-modified staples – I52-30x-DBCO

Staple number	Sequence
2	**/DBCO-TEG/TT**ACATCTGGATGGCGAAATTCTTATATGTGGTAGAGGGTAATA
5	**/DBCO-TEG/TT**CGGTAATGTCACCACATGAAGCCGTCCGAGG
9	**/DBCO-TEG/TT**GGATAGTTAATCAGTACTATGCAGTGCGTAA
11	**/DBCO-TEG/TT**GGATTAGTTAAATATCTTCACGACTCTCGGT
13	**/DBCO-TEG/TT**AAAGATAAAGCTGCGTCGGTCCACTTGCGTA
15	**/DBCO-TEG/TT**GTCAAGTGCGACGATGGACTGTAAAACGACTGATGTGAGTAT
20	**/DBCO-TEG/TT**TGATAACCTGCACACGTCTGGCGGTCAGCAG
22	**/DBCO-TEG/TT**CTGCGGCCCTGCCATAACCATGAGGGTTGTC
24	**/DBCO-TEG/TT**GCGCTTAAGCGCGTAACCACCACTTTACCCT
26	**/DBCO-TEG/TT**GATGGCCCTGCGCTCCTGCAGCGCGTGATCGTCTATCAGGGC
31	**/DBCO-TEG/TT**TTTTTGCAGGAGGACCGAAGGAGCGGGCGCT
32	**/DBCO-TEG/TT**CCGGCGAAATTTAGAGCTTGACGATCATATG
35	**/DBCO-TEG/TT**GATAGGGTATAAATCAAAAGAATGTAAAGCA
36	**/DBCO-TEG/TT**CTTAGACGACTATTAAAGAACGTGGACTCATAATAATGGTTT
40	**/DBCO-TEG/TT**ATGGATGAGCTCATTTTTTAACCAATAGGGAGTCAGGCAACT
44	**/DBCO-TEG/TT**GCTGAATGTCGCCTTGATCGTTGGATGACCA
47	**/DBCO-TEG/TT**CCGGCAACTGGCGAACTACTTACTCTTCATT
48	**/DBCO-TEG/TT**TGTCAGACTCACTGATTAAGCATGGAGCCGG
50	**/DBCO-TEG/TT**CTGATAAAAGATGGTAAGCCCTCCCGTATTGAGACAATAACC
54	**/DBCO-TEG/TT**ACGAGTGGTGCAGGACCACTTATGCGCTCATCAGTTGGGTGC

59	**/DBCO-TEG/TT**GCCTGTAGGCATACACTATTCTCAGAATGGTGACACCACGAT
62	**/DBCO-TEG/TT**TGGCATGATCACAGAAAAGCATCTCATTGTG
64	**/DBCO-TEG/TT**TATCCCGTAAGTTCTGCTATGTGGGAAAGCA
67	**/DBCO-TEG/TT**CGGGTAGGAGCACCTGTATCGGCCGCCCCGA
68	**/DBCO-TEG/TT**AACGCTGGTTCCTGTTTTTGCTCGTGAAGAA
71	**/DBCO-TEG/TT**ACAACAGAAATCTGGCGCCCGGGCGTGTCGC
73	**/DBCO-TEG/TT**CCACCCAATGTGACCTTAAACGAATATTTTT
74	**/DBCO-TEG/TT**CCAAAGATAAGGGCCTCGTGATACGCCTAATAGAATCGGTAT
78	**/DBCO-TEG/TT**TAGGTACACTGAATCTGTCTATACAAACTTTGAGCTGGTTAC
83	**/DBCO-TEG/TT**GCGTTGTTCTAGTCACAGTCTATTATCTTCCTAGATTAGAAG

Supplementary References

1 Shang, J. *et al.* Structural basis of receptor recognition by SARS-CoV-2. *Nature* **581**, 221-224, doi:10.1038/s41586-020-2179-y (2020).

2 Veneziano, R. *et al.* Role of nanoscale antigen organization on B-cell activation probed using DNA origami. *Nat Nanotechnol* **15**, 716-723, doi:10.1038/s41565-020-0719-0 (2020).

3 Knappe, G. A., Wamhoff, E. C., Read, B. J., Irvine, D. J. & Bathe, M. In Situ Covalent Functionalization of DNA Origami Virus-like Particles. *ACS Nano* **15**, 14316-14322, doi:10.1021/acsnano.1c03158 (2021).
