# Supplementary material for: Enhancing antibody responses by multivalent antigen display on thymus-independent DNA origami scaffolds
Source: Nat Commun. 2024 Jan 30;15:795. doi: 10.1038/s41467-024-44869-0 (PMC10828404; doi:10.1038/s41467-024-44869-0)
Supplement: Supplementary file 3 — Reporting Summary [file 41467_2024_44869_MOESM3_ESM.pdf]

nature portfolio

Corresponding author(s): Aaron G. Schmidt; Daniel Lingwood; Mark Bathe

Last updated by author(s): Dec 18, 2023

Reporting Summary

Nature Portfolio wishes to improve the reproducibility of the work that we publish. This form provides structure for consistency and transparency in reporting. For further information on Nature Portfolio policies, see our Editorial Policies and the Editorial Policy Checklist.

Statistics

For all statistical analyses, confirm that the following items are present in the figure legend, table legend, main text, or Methods section.

n/a	Confirmed	
☐	☒	The exact sample size (*n*) for each experimental group/condition, given as a discrete number and unit of measurement
☐	☒	A statement on whether measurements were taken from distinct samples or whether the same sample was measured repeatedly
☐	☒	The statistical test(s) used AND whether they are one- or two-sided<br>*Only common tests should be described solely by name; describe more complex techniques in the Methods section.*
☒	☐	A description of all covariates tested
☐	☒	A description of any assumptions or corrections, such as tests of normality and adjustment for multiple comparisons
☐	☒	A full description of the statistical parameters including central tendency (e.g. means) or other basic estimates (e.g. regression coefficient) AND variation (e.g. standard deviation) or associated estimates of uncertainty (e.g. confidence intervals)
☐	☒	For null hypothesis testing, the test statistic (e.g. *F*, *t*, *r*) with confidence intervals, effect sizes, degrees of freedom and *P* value noted<br>*Give P values as exact values whenever suitable.*
☒	☐	For Bayesian analysis, information on the choice of priors and Markov chain Monte Carlo settings
☒	☐	For hierarchical and complex designs, identification of the appropriate level for tests and full reporting of outcomes
☒	☐	Estimates of effect sizes (e.g. Cohen's *d*, Pearson's *r*), indicating how they were calculated

Our web collection on statistics for biologists contains articles on many of the points above.

Software and code

Policy information about availability of computer code

Data collection	No previously unreported code or software were used.
Data analysis	No previously unreported code or software were used.

For manuscripts utilizing custom algorithms or software that are central to the research but not yet described in published literature, software must be made available to editors and reviewers. We strongly encourage code deposition in a community repository (e.g. GitHub). See the Nature Portfolio guidelines for submitting code & software for further information.

Data

Policy information about availability of data

All manuscripts must include a data availability statement. This statement should provide the following information, where applicable:

- Accession codes, unique identifiers, or web links for publicly available datasets
- A description of any restrictions on data availability
- For clinical datasets or third party data, please ensure that the statement adheres to our policy

The source data used to generate Figs. 1-4 and Supplementary Figs. 1-9 are provided in the Source Data file. Source Data are provided with this paper. The source data are also available from the corresponding authors upon request.

Research involving human participants, their data, or biological material

Policy information about studies with human participants or human data. See also policy information about sex, gender (identity/presentation), and sexual orientation and race, ethnicity and racism.

Reporting on sex and gender	N/A
Reporting on race, ethnicity, or other socially relevant groupings	N/A
Population characteristics	N/A
Recruitment	N/A
Ethics oversight	N/A

Note that full information on the approval of the study protocol must also be provided in the manuscript.

Field-specific reporting

Please select the one below that is the best fit for your research. If you are not sure, read the appropriate sections before making your selection.

☒ Life sciences ☐ Behavioural & social sciences ☐ Ecological, evolutionary & environmental sciences

For a reference copy of the document with all sections, see nature.com/documents/nr-reporting-summary-flat.pdf

Life sciences study design

All studies must disclose on these points even when the disclosure is negative.

Sample size	Power calculations were performed using online software (https://sample-size.net) to determine sample sizes. Based on prior experience we have observed an effect size of ~2.5. Given a need for 80% power to detect an alpha level of 0.05, we required at least 5 experimental units per experimental group.
Data exclusions	No data was excluded.
Replication	We repeated all experiments independently 2x to confirm the reproducibility of our findings. All attempts were successful.
Randomization	Animals were randomly assigned into experimental groups. The mice assigned a number with a random number generator (https://www.random.org) and treatment groups were assigned according to absolute values (e.g top 5 random numbers = treatment 1, bottom 5 random numbers = treatment 2).
Blinding	Structural characterization and cellular experiments for materials were not blinded. For animal experiments, it was not possible to blind immunogen type that went into the animals. The reason is that immunogens were first quality controlled for capacity to trigger BCR signaling in the reporter B cell line and the expertise for BCR signaling and immunization resided in one person.

Reporting for specific materials, systems and methods

We require information from authors about some types of materials, experimental systems and methods used in many studies. Here, indicate whether each material, system or method listed is relevant to your study. If you are not sure if a list item applies to your research, read the appropriate section before selecting a response.

Materials & experimental systems

n/a	Involved in the study	
☐	☒	Antibodies
☐	☒	Eukaryotic cell lines
☒	☐	Palaeontology and archaeology
☐	☒	Animals and other organisms
☒	☐	Clinical data
☒	☐	Dual use research of concern
☒	☐	Plants

Methods

n/a	Involved in the study	
☒	☐	ChIP-seq
☐	☒	Flow cytometry
☒	☐	MRI-based neuroimaging

Antibodies

Antibodies used	Sheep ECL Anti-Human IgG, Horseradish Peroxidase-Linked Species-Specific Whole Antibody (Sigma-Aldrich, Cat No: NA933) final dilution = 1 in 5000.<br>Sheep ECL Anti-mouse IgG, Horseradish peroxidase-linked whole antibody (Sigma-Aldrich, Cat No: NA931) final dilution = 1 in 5000.<br>Goat F(ab')2 Anti-Human IgM-UNLB (SouthernBiotech, Cat No: 2022-01) final dilution = 1 in 100.<br>Goat Anti-Mouse IgM-HRP (SouthernBiotech, Cat. No: 1021-05) final dilution = 1 in 5000.<br>Mouse Anti-Human IgM-HRP (SouthernBiotech, Cat. No: 9020-05) final dilution = 1 in 5000.<br>Mouse Anti-ds DNA antibody-BSA and Azide free (Abcam, Cat No: ab27156, Clone: 35I9) final dilution = 1 in 100.<br>Mouse anti-human IgM APC Antibody (Biolegend, Cat No: 314510, clone: MHM-88) final dilution = 1 in 100.<br>Mouse anti-human-kappa-light chain-PE (ThermoFisher, Cat No: 12-9970-42, Clone TB28-2) final dilution = 1 in 100.<br>Mouse anti-SARS-coV-2 Nucleocapsid (Biolegend; Cat No: 946102; clone: A20087H) final dilution = 1 in 1000.<br>Rat anti-mouse IgG2b-Pe-Cy7 (Biolegend; Cat No: 406714; clone: RMG-2b1) final dilution = 1 in 500.<br>CR3022 human anti-RBD IgG; generated in house; diluted to 200nM<br>B38 human IgG; generated in house; diluted to 200nM
Validation	Sheep ECL Anti-Human IgG, Horseradish Peroxidase-Linked Species-Specific Whole Antibody (Sigma-Aldrich, Cat No: NA933) Manufacturer validation: quality control tested by flow cytometry and immunohisitochemistry. 8 references.<br>Sheep ECL Anti-mouse IgG, Horseradish peroxidase-linked whole antibody (Sigma-Aldrich, Cat No: NA931) Manufacturer validation: quality control tested by flow cytometry and immunohisitochemistry. 8 references.<br>Goat F(ab')2 Anti-Human IgM-UNLB (SouthernBiotech, Cat No: 2022-01) Manufacturer validation: quality control tested by flow cytometry, ELISA and immunohisitochemistry. 15 references.<br>Goat Anti-Mouse IgM-HRP (SouthernBiotech, Cat. No: 1021-05) Manufacturer validation: quality control tested by flow cytometry, ELISA and immunohisitochemistry. 26 references.<br>Mouse Anti-Human IgM-HRP (SouthernBiotech, Cat. No: 9020-05) Manufacturer validation: quality control tested by flow cytometry, ELISA and immunohisitochemistry. 20 references.<br>Mouse Anti-ds DNA antibody-BSA and Azide free (Abcam, Cat No: ab27156, Clone: 35I9) Manufacturer validation: quality control tested by IHC-P and immunohisitochemistry. 99 references.<br>Mouse anti-human IgM APC Antibody (Biolegend, Cat No: 314510, clone: MHM-88) Manufacturer validation: quality control tested by flow cytometry and immunohisitochemistry. 13 references.<br>Mouse anti-human-kappa-light chain-PE (ThermoFisher, Cat No: 12-9970-42, Clone TB28-2) Manufacturer validation: quality control tested by flow cytometry, 3 references.<br>Mouse anti-SARS-coV-2 Nucleocapsid (Biolegend; Cat No: 946102; clone: A20087H) Manufacturer validation: quality control tested by WB, Direct ELISA and ICFC. 3 references.<br>CR3022 human anti-RBD IgG; generated in house; PMID: 32245784<br>B38 human IgG; generated in house; PMID: 32404477

Eukaryotic cell lines

Policy information about cell lines and Sex and Gender in Research

Cell line source(s)	Expi293F cells (human; ThermoFisher; A14527; human)<br>IgM(-) Ramos (human; derived from ATCC CRL-1596 and PMID: 26741406)<br>B38 Ramos (human; this paper and derived from ATCC CRL-1596)<br>CR3022 Ramos (human; this paper and derived from ATCC CRL-1596)<br>ACE2-293T (human; derived from ATCC CRL3216; generously provided by Nir Hacohen and Michael Farzan, Massachusetts General Hospital and The Scripps Research Institute)<br>A549-hAce2 (human; BEI Resources; NR-53821)<br>Vero-E6 (monkey; ATCC; CRL-1586)
Authentication	Ramos B cell IgM surface negative B cell line expressing B38 or CR3022 BCR variants: flow cytometry and plasmid sequencing; authentication of the other commercially obtained cell lines was by morphology
Mycoplasma contamination	All cell lines were negative for mycoplasma contamination.
Commonly misidentified lines<br>(See ICLAC register)	No commonly misidentified cell lines were used in this study.

Animals and other research organisms

Policy information about studies involving animals; ARRIVE guidelines recommended for reporting animal research, and Sex and Gender in Research

Laboratory animals	Wildtype C57Bl/6 mice, Tcra-/- mice, and K18-hACE2 C57BL/6J mice are from Jackson Labs; mice were 6-8 weeks old
Wild animals	No wild animals were used.
Reporting on sex	Females and males were used in this study; this information is disaggregated in the figure captions.

Field-collected samples	No samples were collected in the field.
Ethics oversight	Experiments were approved by institutional animal care and use committee (MGH protocol 2014N000252 and Washington University School of Medicine protocol 21-0246).

Note that full information on the approval of the study protocol must also be provided in the manuscript.

Plants

Seed stocks	N/A
Novel plant genotypes	N/A
Authentication	N/A

Flow Cytometry

Plots

Confirm that:

☒ The axis labels state the marker and fluorochrome used (e.g. CD4-FITC).

☒ The axis scales are clearly visible. Include numbers along axes only for bottom left plot of group (a 'group' is an analysis of identical markers).

☐ All plots are contour plots with outliers or pseudocolor plots.

☒ A numerical value for number of cells or percentage (with statistics) is provided.

Methodology

Sample preparation	For cell binding experiments, a detailed description of the protocol can be found in the methods second "ACE2-expressing cell binding assay". Briefly, ACE-2 expressing HEK 293T cells were harvested and washed with PBS with 2% FBS and 200,000 cells were transferred to 96 well cell culture plates. Cells were incubated with fluorescently labeled RBD constructs for 60 min on ice, washed twice with PBS with 2% FBS, and analyzed.<br><br>For B cell activation experiments, a detailed description of the protocol can be found in the methods second "B cell activation assay". Briefly, IgM expressing Ramos B cells were harvested, incubated with Fura red solution in pre-warmed RPMI for 30 min at 37 °C, spun down, and resuspended in RPMI before analysis. Cells were maintained at 37 °C for the duration of the experiment.
Instrument	BD-LSR-II, Strategim S10000Exi, and Sony Biotech SH800S
Software	FlowJo version 10
Cell population abundance	For cell binding experiments, at least 10,000 events were recorded.<br>For B cell activation assay, data acquisition was obtained for 300 seconds per sample.
Gating strategy	For both cell binding and B cell activation experiments, initial populations were gated using FSC and SSC to remove cell debris and cell clumping. Due to using only this standard gating strategy, no exemplifying figure was provided. This population was then used in fluorescent histograms using either samples stained for the secondary antibody, fluorescent RBD, or ratiometric Fura red signal (unbound versus bound state; em = 657 and 637 nm, respectively).

☐ Tick this box to confirm that a figure exemplifying the gating strategy is provided in the Supplementary Information.
